# A case report of extranodal NK/T-cell lymphoma in patient with chronic lymphocytic leukemia

**DOI:** 10.1097/MD.0000000000011619

**Published:** 2018-07-27

**Authors:** Hangping Ge, Xiangping Wu, Jianping Shen, Junfa Chen, Ying Chen, Yu Zhang

**Affiliations:** a Department of Hematology; b Department of Pathology, First Affiliated Hospital of Zhejiang Chinese Medical University, Hangzhou, China.

**Keywords:** chronic lymphocytic leukemia, extranodal NK/T-cell lymphoma, secondary tumors

## Abstract

**Rationale:**

Chronic lymphocytic leukemia often results in secondary tumors, the most common being large B cell lymphoma known as Richter syndrome, followed by extranodal NK/T-cell lymphoma (nasal type) is extremely rare.

**Patient concerns:**

A chronic lymphocytic leukemia patient presented with nasal congestion.

**Diagnoses:**

Nasal endoscopy identified a left nasal mass, and the pathology suggested extranodal NK/T-cell lymphoma (nasal type).

**Interventions:**

The patient received a course of chemotherapy.

**Outcomes:**

Pneumonia and coagulopathy occurred after chemotherapy, and the patient died shortly thereafter.

**Lessons:**

It is essential to recognize the transformation of disease earlier in chronic lymphoblastic leukemia patient.

## 1 Introduction

Chronic lymphocytic leukemia (CLL) is the most common adult leukemia in Europe and the United States,^[1]^ and a secondary tumor is the most common complication of CLL. The most common form of conversion is large B-cell lymphoma, which is called Richter syndrome.^[2]^ In addition, Hodgkin lymphoma, acute leukemia, multiple myeloma, and other tumors have been observed.^[3]^ We can distinguish whether the transformed tumor and CLL arose from the same clone by testing the mutations in the variable region of the immunoglobulin heavy chain. The factors affecting secondary tumor development include the clinical characteristics of CLL, genetics, and biology. Whether alkylating agents and nucleoside analogues increase the risk of a secondary tumor remains controversial.^[4]^ The prognosis is poor once CLL patients present a secondary tumor. Here, we report a rare case of CLL followed by extranodal NK/T-cell lymphoma (nasal type).

## 2 Case presentation

In 2008, a 69-year-old man presented with increased white blood cell (WBC) (12.6 × 10^9^ cells/L) during a physical examination, whereas the platelets (PLT) and hemoglobin (HB) values were normal. The patient had a fever, night sweats, and weight loss, without superficial lymphadenopathy and hepatosplenomegaly. The patient refused further diagnosis and treatment. In September 2011, the patient underwent a bone marrow puncture that showed 30.5% mature lymphocytes with 4.5% lymphoblasts. The flow cytometry suggested CLL with an abnormal B lymphocyte population accounting for 36.58% of non-erythroid cells and CD5++, CD19+, CD20+, CD23+, HLA-DR+, CD22-, CD38-, sIgMdim, and ZAP-70 expression for 87.6% of CLL cells. The patient declined treatment. In August 2012, routine laboratory results showed WBC 55.7 × 10^9^ cells/L, lymphocyte (LY) 26.9 × 10^9^ cells/L, PLT 69 × 10^9^ cells/L, and HB 144 g/L. Another bone marrow puncture showed 60% mature lymphocytes with 7% lymphoblasts and with the same flow cytometry result; chromosomes: 46, XY; FISH: TP53 gene deletion. B-mode ultrasound examination found multiple enlarged lymph nodes (max 5.3 × 2.3 cm). We diagnosed the patient with CLL (Rai Staging IV). The patient was given chlorambucil (10 mg/m^2^ oral, twice daily from days 1 to 7), followed by 1 course of COP regimen consisting of cyclophosphamide (750 mg/m^2^ i.v. on day 1), vindesine (4 mg i.v. on day 1), and prednisone (60 mg/m^2^ i.v. daily from days 1 to 5), and 1 course of (fludarabine, mitoxantrone, dexaméthasone) FMD regimen consisting of fludarabine (25 mg/m^2^ i.v. daily from days 1 to 3), mitoxantrone (8 mg/m^2^ i.v. on day 1), and dexamethasone (20 mg/m^2^ i.v. daily from days 1 to 5). The WBC decreased to 22.1 × 10^9^ cells/L, and LY decreased to 19.5 × 10^9^ cells/L). Upon follow-up, the patient was in partial remission (PR). From July 2014 to July 2016, the patient underwent one course of FMD regimen consisting of fludarabine (25 mg/m^2^ i.v. daily from days 1 to 3), mitoxantrone (8 mg/m^2^ i.v. on day 1), and dexamethasone (20 mg/m^2^ i.v. daily from days 1 to 5); 2 courses of RFC regimen consisting of rituximab (375 mg/m^2^ i.v. on day 0), fludarabine (25 mg/m^2^ i.v. daily from days 1 to 3), and cyclophosphamide (250 mg/m^2^ i.v. daily from days 1 to 3); and 1 course of RFMD regimen consisting of rituximab (375 mg/m^2^ i.v. on day 0), fludarabine (25 mg/m^2^ i.v. daily from days 1 to 3), mitoxantrone (8 mg/m^2^ i.v. daily on day 1), and dexamethasone (20 mg/m^2^ i.v. daily from days 1 to 5).

**Figure 1:**
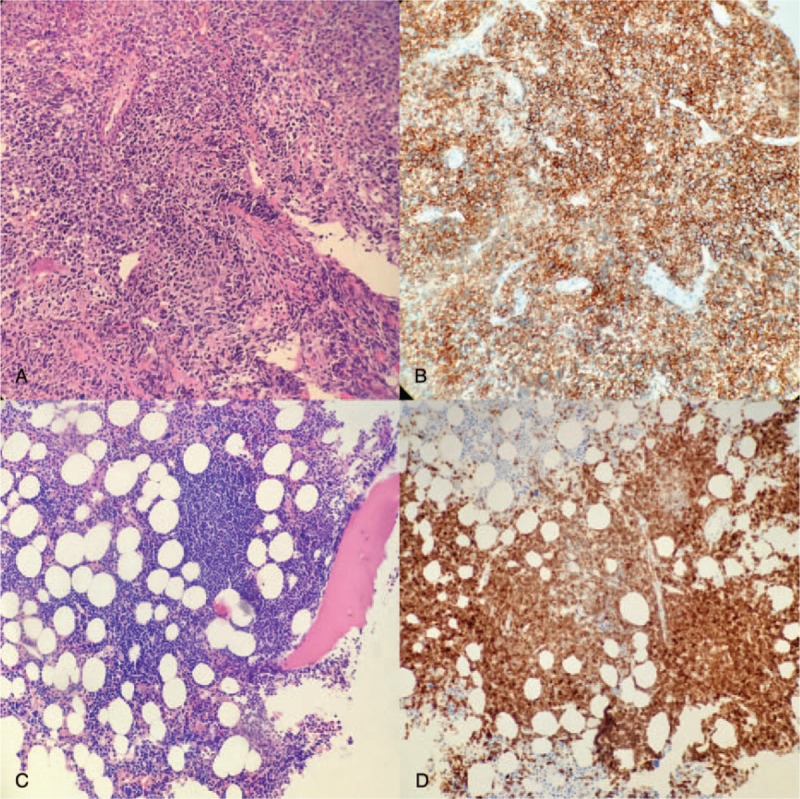
Immunohistochemistry of nasal mass and bone marrow biopsy. (A) hematoxylin-eosin staining of the nasal mass tissue showed a medium-sized cells which have a moderate amount of clear cytoplasm with irregularly folded nuclei, and the cells were CD2+/CD56+/TIA-1+/TCR-; (B) the same cells as A which expressing CD56; (C) hematoxylin-eosin staining of the bone marrow biopsy and the cells are typically small, mature appearing lymphocytes with a dense nucleus, partially aggregated chromatin, and without discernible nucleoli, and were CD19+/CD20+/CD23+/CD5+; (D) the same cells as C which expressing CD5. All images were taken by a SPOT Insight color digital camera at ×20 magnification with a Plan 20×/0.40 objective lens in Olympus microscope.

In May 2017, a nasal endoscopy was performed and revealed a left nasal mass. The pathology suggested extranodal NK/T-cell lymphoma (nasal type, Fig. 1A; CD56-positive, Fig. 1B). The bone marrow biopsy and flow cytometry still confirmed the diagnosis of CLL (Fig. 1C; CD5-positive, Fig. 1D). Gene sequencing of the bone marrow specimen found the *ASXL1* gene had a small segment of insertions/deletions, but no abnormalities were found in the nasal tissue specimen. On May 19^th^, the patient received 1 course of methotrexate, dexamethasone, ifosfamide, Mesna, etoposide, pegaspargase (SMILE) regimen consisting of methotrexate (2 g/m^2^ i.v. on day 1), dexamethasone (40 mg i.v.i.v. daily from days 1 to 4), ifosfamide (1500 mg/m^2^ i.v.i.v. daily from days 2 to 4), Mesna (300 mg/m^2^ i.v. three times a day from days 2 to 4), etoposide (70 mg/m^2^ i.v. three times a day from days 2 to 4), and pegaspargase (2500 U/m^2^ subcutaneously on day 2). Pneumonia and coagulopathy occurred after chemotherapy, and the patient died shortly thereafter.

## 3 Discussion

High-grade lymphoma of the T-cell lineage has been reported only rarely in patients with CLL.^[5,6]^ The presence of a T-cell lymphoma in patients with CLL provides further evidence that Richter syndrome represents the concurrence of 2 independent neoplasms. Our genetic sequencing results also support this view. We suggest that the patient's immunocompromised state provided fertile ground for the development of a T-cell lymphoma. Immune dysfunction combined with chronic antigenic stimulation, perhaps by the CLL cells themselves, allow oligoclonal proliferation of lymphocytes. Epstein-Barr virus (EBV) infection is another factor that may be involved in the development of T-cell lymphoma in patients with CLL. We also identified Epstein-Barr virus-encoded RNA (+) cells in nasal tumor biopsies, and EBV is causally related to nasal-type T-cell lymphoma.^[7]^ The potential that therapy may further increase the risk of a secondary neoplasm is of great concern. Until now, no clear evidence has demonstrated that alkylating agents or purine nucleoside analogs may be associated with an increased incidence of second malignancies in patients with CLL.^[8–10]^ Of course, there are many other risk factors that can lead to secondary tumors or Richter syndrome, including genetic background such as hereditable polymorphisms of BCL2 (rs4987852), CD38 (rs6449182), and low-density lipoprotein receptor-related protein 4 (LRP4; rs2306029); clinical features of CLL such as bulky extensive lymphadenopathy; and biological features of CLL such as the presence of NOTCH1 mutations.^[11]^

There is currently no standard treatment for CLL associated with a secondary tumor or Richter syndrome.^[12,13]^ Generally, the type of disease is determined based on the pathological findings followed by providing appropriate treatment. For example, diffuse large B cell lymphoma (DLBCL) can take an consist of rituximab, cyclophosphamide, vincristine, doxorubicin, dexamethasone (R-CHOP) regimen, platinum-containing regimen or new drug and transplant. The choice of regimen depends on whether the 2 tumors are clonally related.^[14,15]^ The case presented here was an extranodal NK/T-cell lymphoma, which was treated with a first-line SMILE regimen based on the pathologic findings, (comment NO.1), but the patient suffered from infection, coagulopathy, and respiratory failure, and died soon.

## 4 Conclusion

In summary, CLL followed by extranodal NK/T-cell lymphoma (nasal type) is very rare. The occurrence of this secondary tumor is associated with a variety of factors, and the efficacy of traditional treatments is poor. We hope that new drugs can provide more benefit to patients.

## Author contributions

**Conceptualization:** Jianping Shen.

**Data curation:** Xiangping Wu.

**Investigation:** Junfa Chen.

**Project administration:** Yu Zhang.

**Software:** Ying Chen.

**Writing – original draft:** Hangping Ge.
